# 

**Published:** 1887-06-11

**Authors:** 


### THE HOSPITAL

An Institution, Family, and Parochial Journal of Hospitals, Asylums, & all Agencies for the Care of the Sick, Criticism & News.

### CONTENTS.

**Hospital Saturday** - - - - - - 177

**Words of Consolation.**—XXXVI. The Work of the Holy Ghost - - - - - - - 178

**The Archbishop of Canterbury on Hospitals.** 179

**Drugs or No Drugs?** - - - - - - 180

**Counterparts.** By a STUDENT OF EASTERN PHILOSOPHIES. Chap. I - - - - - - - 181

**A National Pension Fund for Hospital Officials and Nurses.**—The Views of Officials, Sisters, Nurses, and Others - - - - - 183

**Notes and News.**—Miscellaneous Hospital News.—Jubilee Celebrations. — The Hospital Campaign.—Hospital Extension in the Isle of Man.—Concert at Grosvenor House.—Hospital Subjects at Burlington House. — Vacancies. — The Royal Family at the Tottenham Hospital.—"Instantaneous" Remedies.—The Seamen's Hospital. — The Poplar Hospital. — Donations, Legacies, etc., to Medical Charities.—Proposed Convalescent Home for Barmouth.— Hospital Reports.— New England Hospital for Women and Children.—Lady Doctors, etc., etc. - - - - 184

**Annotations.** — Co-operation among the Working Classes.—A New Cure for Consumption - - - - 187

**Till the Doctor Comes.**—XX. Treatment of Emergencies - - - - - - - - 188

**A Crusade against Indifference.** Meetings in Aid of the Hospital Sunday Fund - - - - 189

**Chats about Medical Museums.**—By ONE OF THE CROWD —No. VII. King's College - - - 191

**In- and Out-Patients' Hospital Diary** - 192

**The Children's Corner.**—Puzzles, Answers, etc. - vi

**Amusements with Profit** - - - - vi

